# Supplementary material for: Congenital Zika syndrome: A systematic review
Source: PLoS One. 2020 Dec 15;15(12):e0242367. doi: 10.1371/journal.pone.0242367 (PMC7737899; doi:10.1371/journal.pone.0242367)
Supplement: S1 Table — (PDF) [file pone.0242367.s001.pdf]

S1 Table: Systematic review's PICO

Acronym	Criteria	Strategy
(P) population	Pregnant women, newborns and children	"pregnan*" or "children or newborn or infant"
(I) intervention or Exposure	Zika vírus	"Zika or zikv"
(C) comparation	Not avaliable	
(O) outcome	Congenital abnormalities and microcephaly. Microcephaly was included because many published articles did not use references to congenital changes, only microcephaly.	"congenital or congenital abnormalities" or "birth defects or malformations or microcephaly"
