# Supplementary material for: Congenital Zika syndrome: A systematic review
Source: PLoS One. 2020 Dec 15;15(12):e0242367. doi: 10.1371/journal.pone.0242367 (PMC7737899; doi:10.1371/journal.pone.0242367)
Supplement: S1 Fig — (PDF) [file pone.0242367.s002.pdf]

PRISMA 2009 Checklist

Section/topic	#	Checklist item	Reported on page #	Text
**TITLE**				
Title	1	Identify the report as a systematic review, meta-analysis, or both.	Page 1	Congenital Zika Syndrome: A systematic review
**ABSTRACT**				
Structured summary	2	Provide a structured summary including, as applicable: background; objectives; data sources; study eligibility criteria, participants, and interventions; study appraisal and synthesis methods; results; limitations; conclusions and implications of key findings; systematic review registration number.	Page 3	**Background:** The signs and symptoms of the Zika virus infection are mild and self-limited. However, the disease has been linked to ophthalmic and neurologic complications such as Guillain-Barré syndrome, peripheral nerve involvement and to abortion and fetal deaths due to vertical transmission, resulting in various congenital malformations in newborns, such as microcephaly. This review aimed to describe the prevalence of signs and symptoms that characterize the Congenital Zika Syndrome. **Methods and Findings:** A systematic review was performed with a pre-established protocol and described according to the recommendations of the Preferred Reporting Items for Systematic Reviews and Meta-Analyses statement. The search strategy returned 2,048 studies. After the exclusion of duplicates and application of inclusion criteria, 46 studies were included. The main signs and symptoms associated to the congenital zika virus syndrome were microcephaly, abnormal brain findings detected by imaging studies, significant changes in head circumference, low birth weight, low weight according to gestational age, adverse pregnancy outcomes, CNS anomaly, corpus callosal anomaly, cerebral hyperechogenicity and intrauterine fetal death. **Conclusions:** Infection by ZIKV during pregnancy can cause a series of changes in growth and development of children, leading to an impact on the health system by the severity of the cases.Therefore, it is necessary to develop efective and preventive measures such as the use of specific repellents for pregnant women in areas of viral circulation and the establishment of public measures for effective vector control.
**INTRODUCTION**				
Rationale	3	Describe the rationale for the review in the context of what is already known.	Page 3-7	The association of ZIKV infection during pregnancy and congenital malformations has been studied lately due to temporal relationship observed in the last epidemic in Brazil. A causal link between ZIKV and adverse effects during pregnancy is biologically plausible and supported by experimental animal studies at the population and individual levels, although no studies with greater scientific relevance have been found (KRAUER et al., 2017). However, there are still gaps in knowledge about the pathophysiological role of viruses and the definition of all possible signs and symptoms in the effects of ZIKV infection during pregnancy and children throughout development, especially with laboratory confirmation.<br>It is important to mention that most of the published studies correspond to case reports, case series and narrative reviews. These are usually based on early assessments, due investigations developed in the early stages of discovering the association of the virus with a congenital syndrome. Besides, the systematic reviews published address causality, transplacental transmission of ZIKV, the effect of sexual transmission and antibody-dependent enhancement on viral teratogenicity viral.
Objectives	4	Provide an explicit statement of questions being addressed with reference to participants,	Page S7	The review systematic's question is: What are the signs and symptoms associated with Congenital Zika Syndrome? This review had the purpose of knowing the prevalence of signs and symptoms and characterizing the Congenital Zika Syndrome based on a systematic review of the scientific literature.

		interventions, comparisons, outcomes, and study design (PICOS).		
**METHODS**				
Protocol and registration	5	Indicate if a review protocol exists, if and where it can be accessed (e.g., Web address), and, if available, provide registration information including registration number.	Page 8	The study protocol was registered at PROSPERO. The identification number is CRD42020151754.
Eligibility criteria	6	Specify study characteristics (e.g., PICOS, length of follow-up) and report characteristics (e.g., years considered, language, publication status) used as criteria for eligibility, giving rationale.	Page 8	We conducted a systematic review following the PRISMA (Preferred Reporting Items for Systematic Reviews) guidelines. All types of study included:Case reports, case series, cross-sectional studies, cohort studies and case control studies until 02/12/2017. There were no language restrictions in the searches on databases.
Information sources	7	Describe all information sources (e.g., databases with dates of coverage, contact with study authors to identify additional studies) in the search and date last searched.	Page 8	A systematic search was conducted in Medical Literature Analysis and Retrieval System Online (MEDLINE), Latin American and Caribbean Health Sciences Literature, Scientific Electronic Library Online, Web of Science, Excerpta Medica Database (EMBASE), Scopus databases and Biblioteca Virtual em Saúde (BVS) to identify studies assessing signs and symptoms associated to Congenital Zika virus syndrome.
Search	8	Present full electronic search strategy for at least one database, including any limits used, such that it could be repeated.	Page 8	The search descriptors used for MEDLINE were as follows: “zika or zikv” and pregnan*” or “children or newborn or infant” and congenital or congenital abnormalities or birth defects or malformations or microcephaly. The search strategy was adapted according to the characteristics of each database.<br>(((((((zika or zikv)) AND (pregnan* or children or newborn or infant))) AND (congenital or congenital abnormalities or birth defects or malformations or microcephaly)).
Study selection	9	State the process for selecting studies (i.e., screening, eligibility, included in systematic review, and, if applicable, included in the meta-analysis).	Page 8-9	The selection was performed by two authors independently (DFA and WB) and the disagreements were resolved by discussion and consensus. Studies were included if reporting data describing signs and symptoms of fetus growth impairment and altered development in children exposed to Zika virus during pregnancy, among laboratory confirmed mothers and/or children <=2 yo (years-old). First, the titles and abstracts retrieved by the search were read and excluded as follows: editorials, letters, guidelines, and reviews; entomological studies, molecular biology research and in vitro studies; social and psychological research, studies in public policy; descriptive studies on epidemiological profile, research on vaccines and prevention. Secondly, studies potentially eligible for inclusion were read in full text and the same inclusion and exclusion criteria were applied.
Data collection process	10	Describe method of data extraction from reports (e.g., piloted forms, independently, in duplicate) and any processes for obtaining and	Page 9	Data were extracted by one of the authors (DFA) and reviewed by the other (MDW) independently. Discrepancies were resolved by discussion and consensus reached between authors. A standardized data extraction form was elaborated for the review.

		confirming data from investigators.		
Data items	11	List and define all variables for which data were sought (e.g., PICOS, funding sources) and any assumptions and simplifications made.	Page 9 Suplement S1	The form contains 21 items to describe the signs and symptoms presented by fetuses or children, as well as the possible outcomes of pregnancy.<br>This form, available upon request, included the following sections: identification of the study (authors, journal and year of publication, language); studies characteristics (design, total number of patients, period); study population (age, gestational age of infection, diagnostic method, differential diagnosis, pregnancy outcomes, birth weight and head circumference); and analysis method.
Risk of bias in individual studies	12	Describe methods used for assessing risk of bias of individual studies (including specification of whether this was done at the study or outcome level), and how this information is to be used in any data synthesis.	Page 9	Assessment of the methodological quality of the included studies was based on the Methodological Index for Non-randomized Studies (MINORS). The instrument consists of 12 items, the first eight being specific for noncomparative studies. To assess the quality of reports and case series, the Grading of Recommendations, Evaluation, Development and Evaluation (GRADE) was used. The original instrument consists of 8 items, however we used 5 items, because the other are more relevant for cases of adverse drug events, which is not the aim of the articles included in our review (60). Each article was evaluated by two authors (MDW and LG) independently and the disagreements were resolved by consensus.
Summary measures	13	State the principal summary measures (e.g., risk ratio, difference in means).	Page 10	A description of the studies regarding country, study and sample characteristics, clinical examination, imaging exams, necropsy, complementary exams (laboratory, radiography, electrocardiogram) and placental alterations, categorized as neurological alterations, and other physiological systems was performed. The OR and CIs were presented to studies that performed multivariate analysis of the factors associated with zika virus congenital infection (61).
Synthesis of results	14	Describe the methods of handling data and combining results of studies, if done, including measures of consistency (e.g., I^2) for each meta-analysis.	Page 10	The analysis focused on the frequency of the clinical picture described in the papers added to the methodological quality of studies. Give the pre-specified main (most important) outcomes of the review, including details of how the outcome is defined and measured and when these measurement are made, if these are part of the review inclusion criteria.<br>Clinical signs and symptoms, imaging exams, laboratory parameters and necropsy data presented by fetuses and children who were exposed to Zika virus during pregnancy.<br>We aim to describe the main clinical characteristics which may support the definition of the syndrome.

Section/topic	#	Checklist item	Reported on page #	
Risk of bias across studies	15	Specify any assessment of risk of bias that may affect the cumulative evidence (e.g., publication bias, selective reporting within studies).		To avoid the risk of bias, the articles were followed by a protocol and read by two researchers. In case of doubt, a third researcher made the analysis and discussion with the other two.
Additional analyses	16	Describe methods of additional analyses (e.g., sensitivity or subgroup analyses, meta-regression), if done, indicating which were pre-specified.		No further analysis was done.

RESULTS				
Study selection	17	Give numbers of studies screened, assessed for eligibility, and included in the review, with reasons for exclusions at each stage, ideally with a flow diagram.	Page 11, Flowchart S1	The search strategy returned 2,048 studies. After the exclusion of duplicates and application of inclusion criteria in the titles, abstracts and full text, 81 studies were eligible for full text reading. From the full text reading, 46 were included in this review (Flowchart 1).<br>Inclusion of studies:<br>Search strategy (n=2048)<br>Abstract screened (n=162)<br>Titles screened (n=1067)<br>81 Full text (Excluded after full text reading (18 – absence of laboratory confirmed diagnosis; 06 – failed to meet inclusion criteria; 07– no description of signs and symptoms; 04 – same population of other studies included)<br>Total studies included in Review (n=46)
Study characteristics	18	For each study, present characteristics for which data were extracted (e.g., study size, PICOS, follow-up period) and provide the citations.	Page 11-12, Table S1	Most studies were performed in Brazil (59%), epicenter of notified cases, followed by the United States of America (13%) Colombia (9%), French Polynesian (4%) and 2% in Argentina, Eslovênia, Espanha, França, Guiana Francesa, Martinique and Panama (Table 1).
Risk of bias within studies	19	Present data on risk of bias of each study and, if available, any outcome level assessment (see item 12).	Page 11	The quality of articles was assessed by MINORS instrument for all observational studies and by Grading of Recommendations, Assessment, Development and Evaluation (GRADE) approach for case series and case reports. Comparative studies (n=3) achieved good quality criteria, most did not report adequately the following criteria: loss to follow-up less than 5% and prospective calculation of the study size (Figure 1). At least 93% of the reports and case series had excellent quality standards, however 26% did not perform a follow-up long enough for outcomes to occur (Figure 2).
Results of individual studies	20	For all outcomes considered (benefits or harms), present, for each study: (a) simple summary data for each intervention group (b) effect estimates and confidence intervals, ideally with a forest plot.	Page 11-13,15, Table S1	Regarding study design, most of them were case reports (n=22) or case series (n=20); two cohort studies, one case-control and one cross-sectional study.<br>All studies included had at least one laboratory method described for the diagnosis of women and/or children exposed to zika virus during pregnancy. Reverse transcription polymerase chain reaction (RT-PCR) in: serum or urine samples, umbilical cord, placenta, aminiotic fluid, brain and other tissues of the child was the most frequently reported (n=34). Other laboratory diagnosis exams were: IgG (Immunoglobulin G) and IgM (Immunoglobulin M) ELISA Enzyme-linked immunosorbent assay) in 5 articles, and in 25 articles, respectively; plaque reduction neutralization tests (PRNT) in 6 articles and immunohistochemistry of fetal tissues in 3 articles. Most studies (83%) performed differential laboratory diagnosis of other infectious congenital syndromes (syphilis, toxoplasmosis, Rubella, cytomegalovirus, Herpesvirus – STORCH) (Table 1).<br>The gestational age of pregnancy outcomes ranged from 21 to 39 weeks, 66% of them occurred at 38 weeks. Most studies described signs and symptoms of children exposed to zika virus in the first and second trimester (Table 1).<br>Regarding pregnancy outcomes, 36 articles described signs and symptoms of live births, 11 described fetal deaths, 13 miscarriages, 5 neonatal or infant deaths, and 3 described ongoing pregnancy data. The average birth weight ranged from 930g to 3,390g and head circumference ranged from 17 to 35 cm (Table 1).

Synthesis of results	21	Present results of each meta-analysis done, including confidence intervals and measures of consistency.	Page 15-23, Table S2-5	Neurological signs and symptoms were the most frequently described either on imaging exams and autopsy findings. Regarding clinical signs microcephaly was described in twenty-one articles, hypertonicity in eight articles, seizure was described in seven articles, neurological cry / irritability during first months of life reported in six studies (Table 2). Four articles described the association between congenital ZIKV infection, microcephaly and other adverse pregnancy outcomes. One case-control study demonstrated the association of microcephaly and ZIKV tested for Zika virus-specific IgM and by quantitative RT-PCR in serum and cerebrospinal fluid samples of neonates with microcephaly and two controls while maternal serum samples were tested by plaque reduction neutralisation assay for Zika virus and dengue vírus (OR: 55.5 IC 8.6-∞). In addition to microcephaly, De Araújo et al (2016) (21) detected congenital malformations as abnormal brain findings on imaging exams in children with virus in the serum or cerebrospinal fluid (OR: 113.3, IC 14.3- ∞) and serum only (OR: 80.9, IC 10.2-∞), anthropometric changes such as significant changes in head circumference ($p < 0.0001$), birth weight ($p < 0.0001$) and weight according to gestational age ($p<0.0001$). A cohort study conducted in Rio de Janeiro reported that adverse pregnancy outcomes including fetal loss occurred in every trimester ($p < 0.0001$) and need for emergency cesarean section deliveries (17). In a series of cases of children exposed to ZIKV, an association was found between eye changes and infection in the first trimester of pregnancy (OR: 5.1 IC 1.9-13.2), microcephaly (OR: 19, IC 16-61), arthrogryposis (OR: 29, IC 3.3-255.8) and other central nervous system (CNS) injuries (OR: 4.3 IC 1.6-11.2) (62) (Table 3). Infection was associated with CNS anomaly (RR: 2.11 IC 1.18-4.13), corpus callosal anomaly (RR: 2.21 IC 1.08-5.26), cerebral hyperechogenicity (RR: 3.98 IC 1.48-11.49) and intrauterine fetal death (RR: 3.98 IC 1.09-15.17)(41).<br>A frequent clinical sign observed was arthrogryposis reported in twelve articles. Other osteoarticular signs were club foot and hip dysplasia. Morphological changes of the head were clinical signs described in ten articles (overlapping cranial sutures, prominent occipital bone, excess nuchal skin, craniofacial disproportion with depression of frontal and parietal bones); overrinding sutures or closed fontanels were described in five articles.<br>The most frequent ophthalmic changes were in the posterior segment, found in 22% of the reviewed articles that presented abnormalities in the retina (pigmented, chorioretinal atrophy and / or coloboma) and the optic nerve (pallor, atrophy, increased excavation, hypoplasia and / or coloboma) (29,62–67,67,68). Abnormalities of visual function were described in 11% of the articles (63,67–70), as well as extrinsic ocular motility (strabismus (64,68,69) and nystagmus (62,68). Abnormalities in the anterior segment appeared in 9% of the articles (cataracts (64) and glaucoma (66,71) and the refractive error in 4% (astigmatism (68), myopia (66,68) and hyperopia (68) (Table 2).<br>Injuries in other organ systems were also observed to a lesser extent: cardiovascular system (congenital heart disease [n=1]), genitourinary system (ambiguous genitalia [n=1] and bilateral cryptorchidism [n=2]), gastrointestinal system (dysphagia [n=2] (Table 2). Other clinical features were small for gestational age (SGA) in six studies, hearing abnormalities in four, unilateral diaphragmatic paralysis in three articles. Imaging exams were used in 31 (67%) of the articles reviewed (31) to detect altered signs potentially associated to zika virus syndrome: ultrasound (80%), computed tomography (55%), magnetic resonance (29%) and transfontanel ultrasound (23%). Fourteen (45%) and twelve (39%) studies used three and two imaging methods respectively (Table 2). The most frequent signs observed in imaging exams were those from the central nervous system: ventriculomegaly/ increased fluid spaces described in 35 articles; parenchimal or cerebellar calcification (n=33); microcephaly (n=32); hypoplasia or atrophy of brain, cerebellar, brain stem (n=29); abnormal cortical formation (encephalomalacic changes, abnorminal gyration, lissencephaly) (n=27); corpus callosum anomaly (n=18); cysts or pseudocysts (n=18); increased cisterna

				magna (n=7). Other signs described in three articles were: fetal Dandy-walker malformation, hemorrahage and hydrocephaly. Cardiomegaly and diaphragmatic paralysis were detected by chest x-ray (SOUZA et al., 2016b) (Table 4).<br>Additional injuries observed through imaging exams were: poliydraminios or oligohydraminios, intrauterine growth restriction described in seven articles. As the clinical examination indicated, osteoarticular injuries were also frequent: Artrogryposis (n=6) and club foot (n=3). Although less frequent, some other organ systems were involved: gastrointestinal (intestinal hyperechogenicity (n=2), liver calcification and hepatomegaly (n=2), cardiovascular (tachyarrhythmia and cardiomyopathy (n=1), respiratory (hydrothorax (n=1), genitourinary (genitourinary tract anomaly (n=2) (Table 2).<br>Some other injuries observed on imaging exams in a cohort study, which may help the understanding of the natural history of zika virus infection were abnormal middle cerebral artery, abnormal umbilical artery flow and placental insufficiency (BRASIL et al., 2016c) (Table 4).<br>Eight studies (41,50,63,72–76) described the characteristics of the placenta, especially those originating from fetal death. Calcifications in five articles and vascular alterations such as villous infarction and scattered intervillous thrombi in three and two studies respectively.<br>Seven articles described data of miscarriage and necropsy of neonatal and fetal death: microcephaly (n=6), calcifications (n=4), CNS hypoplasia (n=4), ventriculomegaly (n=3) and inflamatory infiltrate in leptomeninges and brain (n=3), arthrogryposis (n=5) and pulmorary hypoplasya (n= 2) (Table 5).<br>One study reported an increase in aspartate antinotransferase and β-2 High Microglobulin and altered microglobulin in seven out of eight children evaluated, while all children presented an increase in ẟ-glutamyl transcriptase and low hemoglobin (42).
Risk of bias across studies	22	Present results of any assessment of risk of bias across studies (see Item 15).		To avoid the risk of bias, the articles were followed by a protocol and read by two researchers. In case of doubt, a third researcher made the analysis and discussion with the other two.
Additional analysis	23	Give results of additional analyses, if done (e.g., sensitivity or subgroup analyses, meta-regression [see Item 16]).		No further analysis was done.
**DISCUSSION**				
Summary of evidence	24	Summarize the main findings including the strength of evidence for each main outcome; consider their relevance to key groups (e.g., healthcare providers, users, and policy makers).	Page 24-29	In our review, the main signs and symptoms associated to the congenital zika virus syndrome were microcephaly, abnormal brain findings detected by imaging studies, significant changes in head circumference, low birth weight and low weight according to gestational age, adverse pregnancy outcomes, CNS anomaly, corpus callosal anomaly, cerebral hyperechogenicity and intrauterine fetal death.<br>Microcephaly was the most frequent sign among the articles in our review. Other studies, using different methodologies, have described similar results (77–79), including increased prevalence of microcephaly among children exposed to ZIKV (56) when compared to the prevalence in other studies of children not exposed to microcephaly, in Brazil and Europe (80,81).<br>Ventriculomegaly was described in 33 studies reviewed (17,41,42,45,45,54,61,61,63–67,69–72,74,76,82–99), an important sign regarding the risk of developing hydrocephalus and the need for surgical intervention for ventriculo-peritoneal shunt, which was also observed in for (100).

				In our study hydrocephalus was related to severe signs and symptoms, such as: epilepsy, motor dysfunction, cognitive dysfunction, arthrogryposis (1/3 of the cases), vision and hearing disorders (50,70,88,101), which is in agreement with the findings of Linden (2019) (100) who identified severe neurological disorders and cognitive dysfunction, no interaction with the environment, absence of motor skills, dysphagia, epilepsy in most cases and arthrogriposis in one third of children who progressed with hydrocephalus. These suggests that hydrocephalus in children exposed to ZIKV appears to be related to other signs and symptoms that indicate the greater severity of the case. Similarly, other congenital infections such as those caused by *Toxoplasma gondii*, Citomegalovirus are also associated with serious brain changes (102,103). However, unlike these, we know that ZIKV has important tropism for fetal neural and ocular progenitor cells and, as a consequence, it can cause, in addition to the hydrocephalus, severe microcephaly with partially collapsed skull, thin brain cortical with subcortical calcifications, macular scars and retinal changes. In addition, Lazear (2016) (104) has seen congenital contractures (bone deformities) less frequently.<br>Parenchimal or cerebellar calcification was a common symptom described among the studies in our review, similar to the characteristics found in congenital infection by congenital cytomegalovirus (CMV), although the distribution of intracranial calcifications is different, that is, typically subcortical in congenital ZIKV infection and periventricular in the CMV (51,105,106).<br>However, it should be noted that the neurological changes of the congenital Zika syndrome have similarities with other syndromes of infectious and also genetic aetiology, such as the Aicardi-Goutières syndrome (107).<br>The osteoarticular system was the second with records of damage to the newborn. Arthrogryposis was the most frequent sign in addition to clubfoot, which was also observed in a recent study that detected a high proportion of children with arthrogriposis, especially the most severe ones (13,17,66,92,100,108,109).<br>Eye changes have been detected in children with microcephaly and other brain changes. However, ZIN (2017) (62)identified that 33% of children with ophthalmic lesions had no CNS abnormalities, suggesting that eye abnormalities may be one of the main findings of the syndrome. It is important to note that as the child's first contact with the environment is through vision, children with visual impairment commonly have delayed neuropsychomotor development, which, in addition to the other signs and symptoms of the syndrome, worsen the child's ability to integrate (110).<br>Ocular changes in the posterior segment of the eye such as macular scarring and pigment mottling have been described as possible features of the severe form of the syndrome (17) as corroborated by Moore et al and Zare et al (111,112). Changes in the anterior segment (cataract and glaucoma) occurred in 9% of the selected studies, which was also observed in a study with children with the syndrome without laboratory confirmation (113).<br>The ocular findings were described in 15 articles of the review as anatomical changes, of these, 5 reported functional changes. However, many of these anatomical changes may impact the child's visual function in the future, as they reach noble areas of the eye (lens, retina and optic nerve). Additionally, Ventura found changes in the posterior segment in 44% of children and abnormality in visual function in 100%, showing that cerebral cortical involvement is also an important cause of visual impairment in these children (68).<br>The pathophysiology of ZIKV infection and the mechanism of passage of the virus through the placental barrier are still being investigated (ROBINSON et al., 2018). The exact timing of placental and fetal infection is also not clear with respect to maternal viremia, as well as the correlation between prolonged viremia and the development of congenital Zika syndrome (57). In our review, placental changes were

				described in 8 studies (41,50,63,72–76), however the role of placental viral infection in determining the severity of the syndrome is not yet defined, as pointed out by other authors who found mild and nonspecific pathological findings of the placenta in pregnancies affected by ZIKV (114–117).<br>Our review shows that the effects of ZIKV on the fetus are more frequent and severe when maternal infection occurs in the 1st and 2nd trimester of pregnancy, resulting in spontaneous abortion; fetal death; therapeutic abortion due to important congenital malformations; several congenital malformations; neonatal and post-neonatal deaths (17,46,62,118) as was also supported in other studies (37,80,119,120). However, it is worth mentioning that the data related to the total number of abortions in Brazil, the epicenter of the cases, may be underestimated, since therapeutic abortion is prohibited by law, although a recent study has not verified an increase in hospitalizations due to abortion complications in the period of the epidemic (121).<br>In addition to adverse pregnancy outcomes and deaths, our review presents evidence that early maternal infection during pregnancy is associated with a greater likelihood of developing congenital abnormalities such as microcephaly, ophthalmic lesions, osteoarticular malformations and other brain changes (41,85) as described in other studies (117,122–132).<br>Among the imaging tests performed, pelvic ultrasound was the most frequent, in addition to transfontanelle USG, computed tomography (CT) and magnetic resonance imaging (MRI) of the newborn (Table 4). Transfontanela USG, CT and MRI were reported less frequently, as they require a specialized team to perform the exam, in addition to the need for sedation of the child.<br>In patients with severe impairment, CZS differs from other congenital infections due to 5 distinct characteristics: (1) severe microcephaly with partially collapsed skull, (2) fine brain corticals with subcortical calcifications, (3) macular scars and focal pigmented retina with spots, (4) congenital contractures and (5) early hypertonia and symptoms of extrapyramidal involvement.<br>Low birth weight and restricted intrauterine growth were common signs in the studies of the present review (42,67,70,76,98,99,108,109,122) representing a risk for children exposed to ZIKV as it was also pointed out in a recent study in which the prevalence of low birth weight (LBW) among children with CZS was more than four times higher when compared to the value found in the sample of live births (133).<br>Disorders in the gastrointestinal system have been described (41,76,108,122,134) including liver disorders, (41,72) intestinal changes, and (72,94) dysphagia. Dysphagia can still increase the risk of bronchoaspiration with consequent aspiration pneumonia or death from asphyxiation and the need for gastrostomy to be installed in the affected children (100,135).<br>Structural changes in the genitourinary system were observed in our study (41,50,134,136). Villamil-gómez (2019) detected severe bilateral renal hypoplasia at necropsy (137); recent studies in older children have detected bladder impairment and possible kidney damage such as very low bladder capacity, bladder hyperactivity with increased consistency, high bladder pressure during the filling phase and high PVR (138).<br>Congenital defects in the cardiovascular system were identified in children exposed to ZIKV (41,97,108,122). Other studies have also described cardiological symptoms such as complex congenital heart disease, echocardiographic abnormalities and cardiac cavity overload (139–142).<br>Pulmonary changes were less common, most detected by necropsy, therefore more severe (50,76,134). Other abnormalities such as hydrothorax (96,143) and diaphragmatic paralysis (50,90,91) have been described. A recent study, evaluating the causality of diaphragmatic paralysis in three infants with CZS, identified phrenic nerve dysfunction detected through electromyography (100).

				Hearing abnormalities have been reported in some studies (42,66,118,122), which points to the need for specific tests for the detection early, as it can further impair the child's interaction with the environment. Hearing impairment was also described by (144).<br>Congenital Zika syndrome and all effects in children, according to the age group, still need to be evaluated in the face of new findings. Our review supported the characterization of the syndrome in children up to 6 months of age, since the onset of the cases. Recent studies have identified neurological disorders in children exposed to ZIKV, asymptomatic at birth (129,145). Such findings are relevant since there is evidence that ZIKV can continue to replicate in the brains of babies after birth (146) and that the brain growth of babies exposed during pregnancy can slow down, even after birth (67).<br>Cohort studies are needed to characterize the signs and symptoms of the syndrome according to the age group to assess the impact on children's cognitive development. Recent studies indicate that neurological changes can impact children's motricity and cognitive level, leading to serious consequences on the social life of the child and their families. Most children have severe impairment of motor function (147,148), delays in functional performance (120), difficulties in feeding and sleeping, abnormalities in vision and hearing, in addition to seizures (148), swallowing dysfunction, movement abnormalities and epilepsy (145). Complications associated with respiratory infection, dysphagia and epilepsy can be fatal for the most affected babies (125,143,149). Congenital Zika syndrome is a serious public health problem, both because of the severity of the cases and the extent of the functional impairment. The possible absence of signs and symptoms at birth does not exclude their late appearance, which presupposes the structuring of health care networks for comprehensive care for children. The high prevalence of asymptomatic cases (65 to 83%) (5,150) may delay the association between congenital syndromes, of late evolution or not, and infection of the mother by ZIKV. Efforts are needed to provide care support for all the needs of children with congenital Zika syndrome and their families, as well as the structuring of health and social care services.
Limitations	25	Discuss limitations at study and outcome level (e.g., risk of bias), and at review-level (e.g., incomplete retrieval of identified research, reporting bias).	Page 30	A limitation of our study was the small number of observational clinical studies. The inclusion of series and case reports, with a lower level of scientific evidence, is justified for the study of new and rare diseases, such as the congenital Zika syndrome, since evidence based on clinical case reports is necessary to understand the illness process (151). In addition, the lack of fetal / neonatal tests in previous studies impaired accurate estimates of maternal-fetal transmission and risk of symptomatic congenital infection. We chose to include only studies with laboratory confirmation of cases, seeking accurate estimates of maternal-fetal transmission and risk of symptomatic congenital infection, which excluded other studies produced during the analysis period.
Conclusions	26	Provide a general interpretation of the results in the context of other evidence, and implications for future research.	Page 31	Infection by ZIKV during pregnancy can cause a series of changes in the growth and development of children, generating an impact on the health system by the severity of the cases and the consequent need for articulation of all levels of complexity of health care and intersectoral agreements for comprehensive care for children. Therefore, it is necessary to develop clinical protocols that include comprehensive family care, as well as the full performance of laboratory tests for STORCH, ZIKV and others. In addition, preventive measures such as the use of specific repellents for pregnant women in areas of viral circulation and the establishment of public measures for effective vector control are essential.
**FUNDING**				

PRISMA 2009 Checklist

Funding	27	Describe sources of funding for the systematic review and other support (e.g., supply of data); role of funders for the systematic review.		Own funding

From: Moher D, Liberati A, Tetzlaff J, Altman DG, The PRISMA Group (2009). Preferred Reporting Items for Systematic Reviews and Meta-Analyses: The PRISMA Statement. PLoS Med 6(7): e1000097. doi:10.1371/journal.pmed1000097

For more information, visit: **www.prisma-statement.org**.
