# Supplementary material for: Congenital Zika syndrome: A systematic review
Source: PLoS One. 2020 Dec 15;15(12):e0242367. doi: 10.1371/journal.pone.0242367 (PMC7737899; doi:10.1371/journal.pone.0242367)
Supplement: S1 Appendix — (PDF) [file pone.0242367.s003.pdf]

UNIVERSITY of York
Centre for Reviews and Dissemination

Systematic review

1. * Review title.

Give the working title of the review, for example the one used for obtaining funding. Ideally the title should state succinctly the interventions or exposures being reviewed and the associated health or social problems. Where appropriate, the title should use the PI(E)COS structure to contain information on the Participants, Intervention (or Exposure) and Comparison groups, the Outcomes to be measured and Study designs to be included.

Congenital Zika Syndrome: A sistematic review

2. Original language title.

For reviews in languages other than English, this field should be used to enter the title in the language of the review. This will be displayed together with the English language title.

Congenital Zika Syndrome: A sistematic review

3. * Anticipated or actual start date.

Give the date when the systematic review commenced, or is expected to commence.

02/12/2017

4. * Anticipated completion date.

Give the date by which the review is expected to be completed.

01/12/2019

5. * Stage of review at time of this submission.

Indicate the stage of progress of the review by ticking the relevant Started and Completed boxes. Additional information may be added in the free text box provided.
Please note: Reviews that have progressed beyond the point of completing data extraction at the time of initial registration are not eligible for inclusion in PROSPERO. Should evidence of incorrect status and/or completion date being supplied at the time of submission come to light, the content of the PROSPERO record will be removed leaving only the title and named contact details and a statement that inaccuracies in the stage of the review date had been identified.
This field should be updated when any amendments are made to a published record and on completion and publication of the review. If this field was pre-populated from the initial screening questions then you are not able to edit it until the record is published.

The review has not yet started: No

Review stage	Started	Completed
Preliminary searches	Yes	No
Piloting of the study selection process	Yes	No
Formal screening of search results against eligibility criteria	Yes	No
Data extraction	Yes	No
Risk of bias (quality) assessment	Yes	No
Data analysis	Yes	No

Provide any other relevant information about the stage of the review here (e.g. Funded proposal, protocol not yet finalised).

6. * Named contact.

The named contact acts as the guarantor for the accuracy of the information presented in the register record.

Danielle Freitas

Email salutation (e.g. "Dr Smith" or "Joanne") for correspondence:

Ms Freitas

7. * Named contact email.

Give the electronic mail address of the named contact.

dafufrj@gmail.com

8. Named contact address

Give the full postal address for the named contact.

Avenida Dom Helder Câmara, 6001 - Bloco 4 , Apartamento 205 - Pilares CEP: 20771002

9. Named contact phone number.

Give the telephone number for the named contact, including international dialling code.

+5521973225393

10. * Organisational affiliation of the review.

Full title of the organisational affiliations for this review and website address if available. This field may be completed as 'None' if the review is not affiliated to any organisation.

Fundação Oswaldo Cruz

Organisation web address:

Rua Leopoldo Bulhões, 1480 –Térreo - Manguinhos - Rio de Janeiro – RJ - CEP: 21041-210

11. * Review team members and their organisational affiliations.

Give the personal details and the organisational affiliations of each member of the review team. Affiliation refers to groups or organisations to which review team members belong. **NOTE: email and country are now mandatory fields for each person.**

Ms Danielle Freitas. Fundação Oswaldo Cruz
Dr Reinaldo Santos. Fundação Oswaldo Cruz
Dr Mayumi Wakimoto. Fundação Oswaldo Cruz
Ms Liege Carvalho. Fundação Oswaldo Cruz
Mrs Luiza Neves. Fundação Oswaldo Cruz
Dr Andrea Zin. Fundação Oswaldo Cruz

12. * Funding sources/sponsors.

Give details of the individuals, organizations, groups or other legal entities who take responsibility for initiating, managing, sponsoring and/or financing the review. Include any unique identification numbers assigned to the review by the individuals or bodies listed.

Own funding

Grant number(s)

13. * Conflicts of interest.

List any conditions that could lead to actual or perceived undue influence on judgements concerning the main topic investigated in the review.

None

14. Collaborators.

Give the name and affiliation of any individuals or organisations who are working on the review but who are not listed as review team members. **NOTE: email and country are now mandatory fields for each person.**

15. * Review question.

State the question(s) to be addressed by the review, clearly and precisely. Review questions may be specific or broad. It may be appropriate to break very broad questions down into a series of related more specific questions. Questions may be framed or refined using PI(E)COS where relevant.

What are the signs and symptoms associated with Congenital Zika Syndrome?

16. * Searches.

State the sources that will be searched. Give the search dates, and any restrictions (e.g. language or publication period). Do NOT enter the full search strategy (it may be provided as a link or attachment.)

We conducted a systematic review using as sources the following data base: PubMed/MEDLINE, Web of Science, Embase, SciELO, LILACS, Biblioteca Virtual em Saúde (BVS) and Scopus.

There were no restrictions of publication period or languages

We conducted a systematic review following the PRISMA (Preferred Reporting Items for Systematic Reviews) guidelines.

17. URL to search strategy.

Give a link to a published pdf/word document detailing either the search strategy or an example of a search strategy for a specific database if available (including the keywords that will be used in the search strategies), or upload your search strategy.Do NOT provide links to your search results.

(((((((zika or zikv)) AND (pregnan* or children or newborn or infant))) AND (congenital or congenital abnormalities or birth defects or malformations or microcephaly)).

Alternatively, upload your search strategy to CRD in pdf format. Please note that by doing so you are consenting to the file being made publicly accessible.

Do not make this file publicly available until the review is complete

18. * Condition or domain being studied.

Give a short description of the disease, condition or healthcare domain being studied. This could include health and wellbeing outcomes.

The association of ZIKV infection during pregnancy and congenital malformations has been studied lately due to temporal relationship observed in the last epidemic in Brazil. A causal link between ZIKV and adverse effects during pregnancy is biologically plausible and supported by experimental animal studies at the population and individual levels, although no studies with greater scientific relevance have been found (KRAUER et al., 2017). However, there are still gaps in knowledge about the pathophysiological role of viruses and the definition of all possible signs and symptoms in the effects of ZIKV infection during pregnancy and children throughout development, especially with laboratory confirmation.

19. * Participants/population.

Give summary criteria for the participants or populations being studied by the review. The preferred format includes details of both inclusion and exclusion criteria.

We included all studies containing description of signs and symptoms presented by fetuses or children who were exposed to ZIKV during pregnancy of laboratory confirmed cases.

Exclusion criteria: reviews, protocols and guides; animal and sector research; studies in public policy and popular actions; social and psychological research; in vitro studies ; studies of vaccines and prevention.

20. * Intervention(s), exposure(s).

Give full and clear descriptions or definitions of the nature of the interventions or the exposures to be reviewed.

Laboratory confirmed ZIKV infection in pregnant women and children.

21. * Comparator(s)/control.

Where relevant, give details of the alternatives against which the main subject/topic of the review will be compared (e.g. another intervention or a non-exposed control group). The preferred format includes details of both inclusion and exclusion criteria.

Laboratory confirmed ZIKV infection in pregnant women and children.

22. * Types of study to be included.

Give details of the types of study (study designs) eligible for inclusion in the review. If there are no restrictions on the types of study design eligible for inclusion, or certain study types are excluded, this should be stated. The preferred format includes details of both inclusion and exclusion criteria.

Case reports, case series, cross-sectional studies, cohort studies and case control studies.

23. Context.

Give summary details of the setting and other relevant characteristics which help define the inclusion or exclusion criteria.

24. * Main outcome(s).

Give the pre-specified main (most important) outcomes of the review, including details of how the outcome is defined and measured and when these measurement are made, if these are part of the review inclusion criteria.

Give the pre-specified main (most important) outcomes of the review, including details of how the outcome is defined and measured and when these measurement are made, if these are part of the review inclusion criteria.

Clinical signs and symptoms, imaging exams, laboratory parameters and necropsy data presented by fetuses and children who were exposed to Zika virus during pregnancy.

We aim to describe the main clinical characteristics which may support the definition of the syndrome.

* Measures of effect

Please specify the effect measure(s) for you main outcome(s) e.g. relative risks, odds ratios, risk difference, and/or 'number needed to treat.

Not applicable

25. * Additional outcome(s).

List the pre-specified additional outcomes of the review, with a similar level of detail to that required for main outcomes. Where there are no additional outcomes please state 'None' or 'Not applicable' as appropriate to the review

Not applicable

* Measures of effect

Please specify the effect measure(s) for you additional outcome(s) e.g. relative risks, odds ratios, risk difference, and/or 'number needed to treat.

Not applicable

26. * Data extraction (selection and coding).

Describe how studies will be selected for inclusion. State what data will be extracted or obtained. State how this will be done and recorded.

The selection of studies was performed by two independent observers and another researcher participated to solve disagreements by discussion and consensus.

Data extraction was performed using the form elaborated for the study. The form contains 21 items to

describe the signs and symptoms presented by fetuses or children, as well as the possible outcomes of pregnancy.

27. * Risk of bias (quality) assessment.

Describe the method of assessing risk of bias or quality assessment. State which characteristics of the studies will be assessed and any formal risk of bias tools that will be used.

Assessment of the methodological quality of the studies was based on the the methodological index for non-randomized studies (MINORS) (SLIM et al., 2003).

28. * Strategy for data synthesis.

Provide details of the planned synthesis including a rationale for the methods selected. This **must not be generic text** but should be **specific to your review** and describe how the proposed analysis will be applied to your data.

The folllowing data were analyzed: type of study, laboratory diagnostic method, differential TORCH test, gestational outcomes, mean age of the mother, gestational age of infection, gestational age of pregnancy outcome, weight and head circumference of children, laboratory parameters, imaging exams, necropsy data. They were categorized and summarized so that the frequency of citation in the studies could be clear. The analysis focused on the frequency of the clinical picture described in the papers added to the methodological quality of studies.

29. * Analysis of subgroups or subsets.

State any planned investigation of ‘subgroups’. Be clear and specific about which type of study or participant will be included in each group or covariate investigated. State the planned analytic approach.

None planned

30. * Type and method of review.

Select the type of review and the review method from the lists below. Select the health area(s) of interest for your review.

Type of review

Cost effectiveness
No

Diagnostic
Yes

Epidemiologic
No

Individual patient data (IPD) meta-analysis
No

Intervention
No

Meta-analysis
No

Methodology
No

Narrative synthesis
No

Network meta-analysis
No

Pre-clinical
No

Prevention
No

Prognostic
No

Prospective meta-analysis (PMA)
No

Review of reviews
No

Service delivery
No

Synthesis of qualitative studies
No

Systematic review
Yes

Other
No

Health area of the review

Alcohol/substance misuse/abuse
No

Blood and immune system
No

Cancer
No

Cardiovascular
No

Care of the elderly
No

Child health
Yes

Complementary therapies
No

COVID-19
No

Crime and justice
No

Dental
No

Digestive system
No

Ear, nose and throat
No

Education

No

Endocrine and metabolic disorders
No

Eye disorders
No

General interest
No

Genetics
No

Health inequalities/health equity
No

Infections and infestations
Yes

International development
No

Mental health and behavioural conditions
No

Musculoskeletal
No

Neurological
No

Nursing
No

Obstetrics and gynaecology
No

Oral health
No

Palliative care
No

Perioperative care
No

Physiotherapy
No

Pregnancy and childbirth
Yes

Public health (including social determinants of health)
No

Rehabilitation
No

Respiratory disorders
No

Service delivery
No

Skin disorders
No

Social care
No

Surgery
No

Tropical Medicine
Yes

Urological
No

Wounds, injuries and accidents
No

Violence and abuse
No

31. Language.

Select each language individually to add it to the list below, use the bin icon to remove any added in error.
English

There is an English language summary.

32. * Country.

Select the country in which the review is being carried out from the drop down list. For multi-national collaborations select all the countries involved.

Brazil

33. Other registration details.

Give the name of any organisation where the systematic review title or protocol is registered (such as with The Campbell Collaboration, or The Joanna Briggs Institute) together with any unique identification number assigned. (N.B. Registration details for Cochrane protocols will be automatically entered). If extracted data will be stored and made available through a repository such as the Systematic Review Data Repository (SRDR), details and a link should be included here. If none, leave blank.

34. Reference and/or URL for published protocol.

Give the citation and link for the published protocol, if there is one

Give the link to the published protocol.

Alternatively, upload your published protocol to CRD in pdf format. Please note that by doing so you are consenting to the file being made publicly accessible.

No I do not make this file publicly available until the review is complete

Please note that the information required in the PROSPERO registration form must be completed in full even if access to a protocol is given.

35. Dissemination plans.

Give brief details of plans for communicating essential messages from the review to the appropriate audiences.

Post graduate program of the “Escola Nacional de Saúde Pública – Fiocruz”, scientific conferences, scientific publications.

Do you intend to publish the review on completion?

Yes

36. Keywords.

Give words or phrases that best describe the review. Separate keywords with a semicolon or new line. Keywords will help users find the review in the Register (the words do not appear in the public record but are

included in searches). Be as specific and precise as possible. Avoid acronyms and abbreviations unless these are in wide use.

Zika virus infection; Zika virus; Humans;Female; Pregnancy;Fetus; Infant; congenital abnormalities: birth defects and malformations.

37. Details of any existing review of the same topic by the same authors.

Give details of earlier versions of the systematic review if an update of an existing review is being registered, including full bibliographic reference if possible.

None

38. * Current review status.

Review status should be updated when the review is completed and when it is published. For newregistrations the review must be Ongoing.
Please provide anticipated publication date

Review_Ongoing

39. Any additional information.

Provide any other information the review team feel is relevant to the registration of the review.

None

40. Details of final report/publication(s) or preprints if available.

This field should be left empty until details of the completed review are available OR you have a link to a preprint.

Give the link to the published review.
