# Supplementary material for: Congenital Zika syndrome: A systematic review
Source: PLoS One. 2020 Dec 15;15(12):e0242367. doi: 10.1371/journal.pone.0242367 (PMC7737899; doi:10.1371/journal.pone.0242367)
Supplement: S2 Appendix — (PDF) [file pone.0242367.s004.pdf]

Systematic review studies' quality assessment

Quality assessment of the studies included in the systematic review based on Methodological Index for Non-Randomized Studies (MINORS).

Studies	MINOR instrument issues											
	1 - A clearly stated aim	2 - Inclusion of consecutive patients	3 - Prospective collection of data	4 - Endpoints appropriate to the aim of the study	5 - Unbiased assessment of the study endpoint	6 - Follow-up period appropriate to the aim of study	7 - Loss to follow-up less than 5%	8 - Prospective calculation of the study size	9 - An adequate control group	10 - Contemporary groups	11 - Baseline equivalence of groups	12 - Adequate statistical analyses
Schaub et al (2017) [58]	1	2	1	2	2	2	0	0	0	0	0	0
Carvalho et al (2016) [59]	2	2	2	2	2	2	0	0	0	0	0	0
Pomar et al (2017)[53]	2	2	2	2	2	1	1	2	2	0	2	2
de Araújo et al (2016) [20]	2	2	2	2	2	2	2	2	2	0	2	2
Honein et al (2017) [54]	2	2	2	2	2	2	1	0	0	0	0	0
Aragão et al (2017) [60]	2	2	0	2	2	2	0	0	0	0	0	0
Hazin et al (2016) [61]	1	1	0	2	2	2	0	0	0	0	0	0
Oliveira-Szejnfeld et al (2016) [62]	2	2	2	2	2	2	0	0	0	0	0	0
Besnard et al (2016) [63]	2	2	0	2	2	2	0	0	0	0	0	0
Van der Linden et al (2017) [66]	2	2	2	2	2	2	0	0	0	0	0	0
Melo et al (2016) [65]	2	2	2	2	2	2	2	2	0	0	0	0
van der Linden et al (2016) [64]	2	2	0	2	2	2	0	0	0	0	0	0
Meneses et al (2017) [68]	2	1	1	1	1	2	0	0	0	0	0	0
Sousa et al (2017) [70]	2	2	2	2	2	2	0	0	0	0	0	0
Castro et al (2017) [72]	2	2	2	2	2	2	0	0	0	0	0	0
Ventura et al (2017) [57]	2	2	1	2	2	2	0	0	0	0	0	0
Zin et al (2017) [55]	2	1	0	2	2	2	0	0	0	0	0	0
Parra-Saavedra et al (2017) [74]	1	1	1	1	1	1	0	0	0	0	0	0
Aragão et al (2017)[56]	2	2	2	2	2	2	0	0	2	0	2	2
Del Campo et al (2017) [75]	2	2	2	2	2	2	0	0	0	0	0	0
Chimelli et al (2017) [76]	2	2	2	2	2	2	0	0	0	0	0	0
Schaub et al (2017) [77]	2	2	2	2	2	2	0	0	0	0	0	0
Ventura et al (2016)[73]	2	1	2	2	2	2	0	0	0	0	0	0
Brasil et al (2016)[16]	2	2	2	2	2	2	1	2	2	0	2	2

Reported and adequate: 2; Reported but inadequate: 1; Not reported: 0.

Quality assessment of the studies included in the systematic review based on JBI (Joanna Briggs Institute) critical appraisal checklist for case reports.

Studies	JBI instrument issues							
	1 - Were patient's demographic characteristics clearly described?	2 - Was the patient's history clearly described and presented as a timeline?	3 - Was the current clinical condition of the patient on presentation clearly described?	4 - Were diagnostic tests or assessment methods and the results clearly described?	5 - Was the intervention(s) or treatment procedure(s) clearly described?	6 - Was the post-intervention clinical condition clearly described?	7 - Were adverse events (harms) or unanticipated events identified and described?	8 - Does the case report provide takeaway lessons?
Mattar et al (2017) [78]	2	2	2	2	2	0	0	2
Culjat et al (2016)[79]	2	2	2	2	2	0	0	2
Souza et al (2016) [80]	2	1	2	2	2	0	0	2
Perez et al (2016) [81]	2	2	2	2	2	0	0	2
Van der Linden et al (2017) [82]	2	2	2	2	2	0	0	2
Souza et al (2016)[83]	2	2	2	2	2	0	0	2
Vorona et al (2016) [84]	2	1	1	2	2	0	0	2
Sanín-Blair et al (2017) [67]	2	2	2	2	2	0	0	2
Zacharias et al (2017) [85]	2	2	2	2	2	0	0	2
Werner et al (2016) [86]	2	2	2	2	2	0	0	2
Freitas et al (2016) [87]	2	2	2	2	2	0	0	2
Moron et al (2016)[88]	1	2	2	2	2	0	0	2
Frutos et al (2017) [89]	2	1	2	2	2	0	0	2
Fernandez et al (2017) [69]	2	1	2	2	2	0	0	2
Martines et al (2016) [35]	2	1	2	2	2	0	0	2
Guillemette-Artur et al (2016) [71]	2	2	2	2	2	0	0	2
Acosta-Reyes et al (2017) [90]	2	2	2	2	2	0	0	2
Vesnaver et al (2016) [91]	2	1	2	2	2	0	0	2
Sarno et al (2016) [92]	2	2	2	2	2	0	0	2
Driggers et al (2016) [40]	2	1	2	2	2	0	0	2
Oliveira et al (2016) [93]	2	1	2	2	2	0	0	2
Narero et al (2016) [94]	2	2	2	2	2	0	0	2

Yes:2; No:1; Not applicable: 0.
