# Supplementary figures and images for: Congenital Zika syndrome: A systematic review
Source: PLoS One. 2020 Dec 15;15(12):e0242367. doi: 10.1371/journal.pone.0242367 (PMC7737899; doi:10.1371/journal.pone.0242367)

S3 Appendix: Search strategy

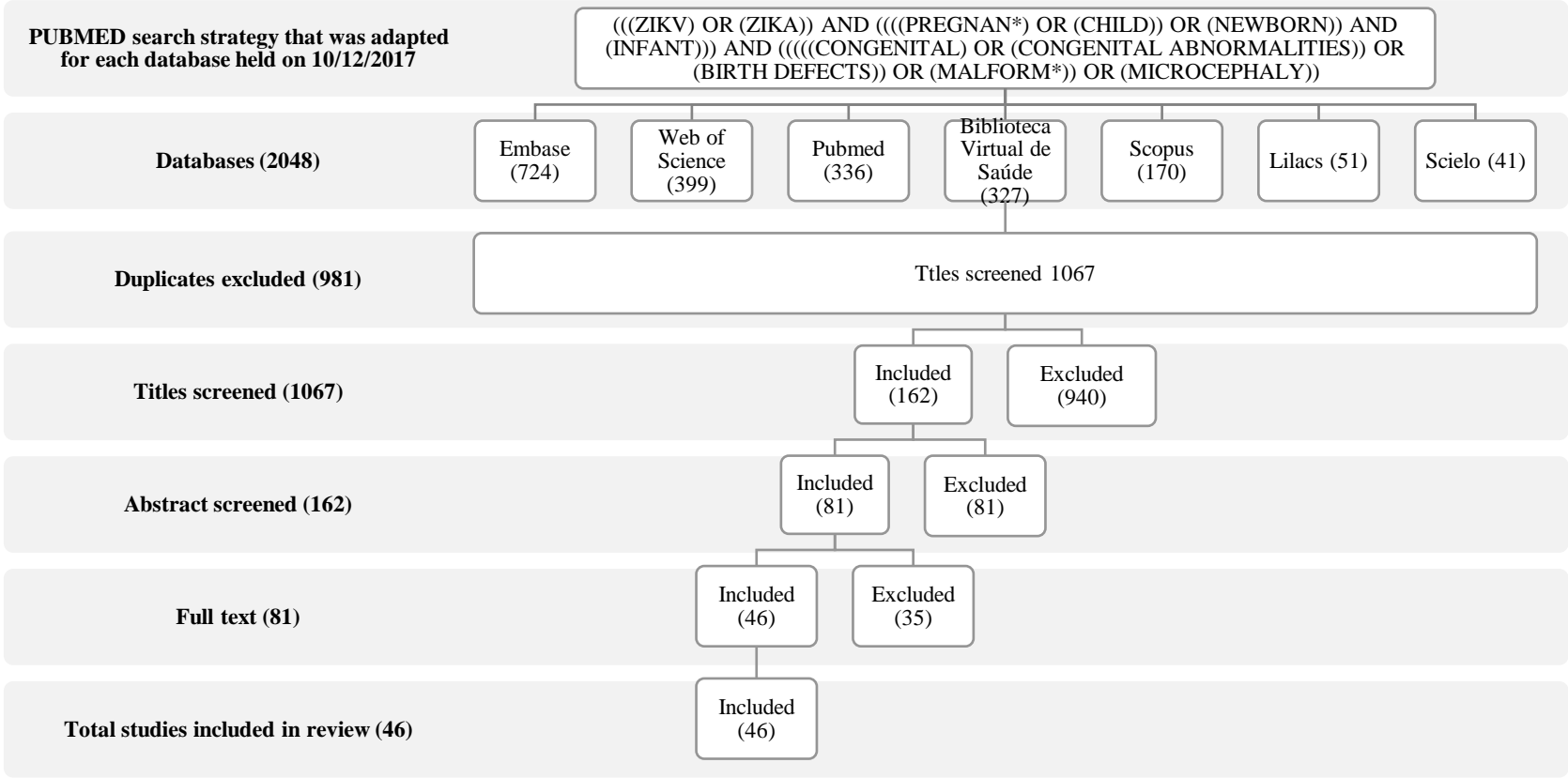

Supplement: S3 Appendix — (PDF) [file pone.0242367.s005.pdf]
