# Supplementary material for: Congenital Zika syndrome: A systematic review
Source: PLoS One. 2020 Dec 15;15(12):e0242367. doi: 10.1371/journal.pone.0242367 (PMC7737899; doi:10.1371/journal.pone.0242367)
Supplement: S1 File — (PDF) [file pone.0242367.s006.pdf]

S1 File

Table 1: Neurological disorders.

Signs	d Diagnostic method[*]	Children exposed to ZIKV			Studies (N=46)
		Min[δ]	Max[£]	n (%)	
Microcephaly	Image,, Clinical, Autopsy	1	87	41 (89)	[16,37,42,55,56,57,59,60,61,62,63,64,66,67,68,70,71,72,73,75,76,77,78,79–84,86,87,88,89,90,91,93–98]
Parenchymal or cerebellar calcification	Image,, Autopsy	1	81	37 (80)	[16,20,37,42,56,59–68,70,71,72,73,75–77,78,79–82,84,87,88,90,91,93–95,97,98]
Ventriculomegaly/ increased fluid spaces	Image,, Autopsy	3	81	34 (83)	[16,20,37,42,55,61–68,70,71,72,73,75–77,78,79–82,84,86–91,93,95,97,98]
Hypoplasia or atrophy of cerebral cortex, cerebellum, brainstem	Image,, Autopsy	1	41	32 (69)	[16,37,42,59–64,66–68,70,71,72,73,76,77,78,79,81,82,84,86,88,90–93,95,97,98]
Abnormal cortical formation (encephalomalacic changes, abnormal gyration, lissencephaly)	Image,, Autopsy	1	81	27 (59)	[16,20,37,55,62–64,66–68,70,71,72,73,75–79,81,84,87–90,97,98]
Corpus callosum anomaly	Image,	1	32	18 (39)	[42,55,59,62,64–67,71,73,76,77,79,88,89,91,93,95]
Cysts or pseudocysts	Image,	1	41	8 (17)	[16,60,65,73,84,85,93,98]
Hypertonicity	Clinical	1	58	8 (17)	[16,59,60,68,77,84,90,91]
Seizures	Clinical	1	87	7 (15)	[16,37,59,67,68,70,81]
Increased cisterna magna	Image,	1	41	7 (15)	[16,62,66,82,90,93,95]
Neurological crying/irritability during first months of life	Clinical	1	58	6 (13)	[16,68,77,80,84,91]
Hyperexcitability/hyperreflexia	Clinical	1	58	5 (11)	[16,67,77,81,84]
Asymmetrical tonic neck reflex	Clinical	1	13	4 (9)	[67,68,80,84]
Fetal Dandy-Walker malformation	Image,	1	30	3 (7)	[61,70,87]
Hemorrhage	Image,	1	41	3 (7)	[16,42,93]
Hydrocephalus	Image, Autopsy	1	8	3 (7)	[37,60,83]
Inflammatory infiltrate in leptomeninges and brain	Autopsy	1	7	3 (7)	[37,42,72]
Vascular congestion in leptomeninges or brain	Autopsy	3	7	2 (4)	[37,72]
Clenched fists	Clinical	12	58	2 (4)	[16,77]
Distal tremors	Clinical	12	58	2 (4)	[16,77]
Centrally decreased muscle tone (upper extremities)	Clinical	1	58	2 (4)	[16,81]
Neurological impairment	Clinical	1	58	2 (4)	[16,80]
Encephalocele	Clinical	1	26	2 (4)	[56,96]
Altered visual fixation and pursuit	Clinical	2	13	2 (4)	16-17
Posterior fossa anomaly	Image,	14	27	2 (4)	[55,79]
Cerebellar abnormalities	Image,	17	24	2 (4)	[75,76]
Anencephaly	Image,	2	4	2 (4)	[71,92]
Lenticulostriate vasculopathy	Image,	2	2	2 (4)	[84,85]
Supratentorial dilatation	Image,			1 (2)	[16]
Ischemic parenchymal lesions	Image,			1 (2)	[16]
Abnormal middle cerebral artery	Image,			1 (2)	[16]
Brachycephaly	Image,			1 (2)	[16]
Cerebral hyperechogenicity	Image,			1 (2)	[55]
Abnormal pons	Image,			1 (2)	[79]
Polymalformative syndrome (encephalocele, anophthalmia, arthrogryposis, fetal hydrops)	Image,			1 (2)	[79]
Holoprosencephaly	Image,			1 (2)	[71]
Schizencephaly	Image,			1 (2)	[92]
Hydranencephaly	Image,			1 (2)	[94]
Encephalocele	Image,			1 (2)	[96]
Colpocephaly	Image,			1 (2)	[88]
Abnormal posturing	Clinical			1 (2)	[16]
Altered motor reflexes	Clinical			1 (2)	[16]
Hemiparesis	Clinical			1 (2)	[16]
Hypoactivity	Clinical			1 (2)	[16]
Hydrocephalus	Clinical			1 (2)	[83]
Cortical blindness	Clinical			1 (2)	[81]
Anterior spinal horn cell loss	Autopsy			1 (2)	[78]
Cerebral gliosis	Autopsy			1 (2)	[72]
Holoprosencephaly	Autopsy			1 (2)	[37]
Cerebral necrosis	Autopsy			1 (2)	[37]
Hydrocephalus	Autopsy			1 (2)	[37]
Dandy-Walker syndrome	Autopsy			1 (2)	[87]

Table 2: Osteoskeletal abnormalities.

Signs	Diagnostic method *	Children exposed to ZIKV			Studies (N=46)
		Min^ð	Max^£	n (%)	
Arthrogryposis	Clinical	1	87	18(39)	[16,37,58,61,66,67,68,70,71,72,77,78,82–84,87,89,94]
Clubfoot	Clinical, Image	1	41	5 (11)	[82,84] [16,61,76]
Hip dysplasia	Clinical	13	58	2 (4)	[16,68]
Knee fovea	Clinical			1 (2)	[16]
Cortical thumb	Clinical			1 (2)	[16]
Elbow fovea	Clinical			1 (2)	[16]
Polydactyly	Clinical			1 (2)	[16]
Hand contractures / Camptodactyly	Clinical			1 (2)	[77]
Feet malposition / contractures	Clinical			1 (2)	[77]
Prominent calcaneus	Clinical			1 (2)	[77]

Table 3: Ophthalmic abnormalities.

Signs		Diagnostic method *	Children exposed to ZIKV			Studies (N=46)
			Min^ð	Max^£	n (%)	
Posterior segment abnormalities	Retinal abnormalities &	Clinical	1	16	10(22)	[57,59,66–68,70,75,81,84,89]
	Optic nerve abnormalities#	Clinical	1	15		[57,59,66,67,75,81,84,89]
Abnormal visual function		Clinical	1	32	5 (11)	[59,60,68,77,81]
Extrinsic eye motility	Strabismus	Clinical	¨	30	5 (11)	[59,67,77] ¨
	Nystagmus	Clinical	6	9		[57,59]
Anterior segment abnormalities	Cataract	Clinical	1	1	4 (9)	[67,95]
	Glaucoma	Clinical	1	1		[84,89]
Refractive error	Astigmatism	Clinical			2 (4)	[59]
	Myopia	Clinical				[59,84]
	Hyperopia	Clinical				[59]

Table 4: Abnormalities in other systems.

Signs	Diagnostic method*	Children exposed to ZIKV		Studies (N=46)	
		Min$^{\delta}$	Max$^{£}$	n (%)	
Morphological changes of the headΦ	Clinical	1	87	10 (22)	[16,37,62,66,68,70,77,80,81,84]
Intrauterine growth restriction	Image, Autopsy	1	41	8 (17)	[16,60,61,72,79,82,88,94]
Small for gestational age (SGA)	Clinical, Image	1	58	8 (17)	[16,20,37,55,66,81,82,87]
Polyhydramnios/oligohydramnios	Image	1	41	7 (15)	[16,55,61,67,71,87,97]
Overriding sutures or closed fontanels	Clinical	1	13	5 (11)	[67,68,77,80,81]
Hearing abnormalities	Clinical	1	58	4 (9)	[16,56,60,84]
Unilateral diaphragmatic paralysis	Clinical	3	87	3 (7)	[37,67,70]
Premature closure of fontanelle	Image	1	6	3 (7)	[90,62,88]
Pulmonary hypoplasia	Autopsy	1	3	3 (7)	[37,72,87]
Hepatomegaly/liver calcifications	Image	8	27	3 (7)	[55,60]
Liver/spleen echogenicity	Image	27	41	2 (4)	[16,55]
Intestinal hyperechogenicity	Image	4	27	2 (4)	[55,65]
Genitourinary tract anomaly	Image	3	27	2 (4)	[37,55]
Subcutaneous edema	Image	1	1	2 (4)	[93,94]
Hydrothorax	Image	1	1	2 (4)	[93,94]
Dystonic movement	Clinical	32	58	2 (4)	[16,59]
Dysphagia	Clinical	13	58	2 (4)	[16,68]
Bilateral cryptorchidism.	Clinical	1	3	2 (4)	[37,81]
Pulmonary hemorrhage	Autopsy	3	7	2 (4)	[37,72]
Virus detected in fetal tissues	Autopsy	3	7	2 (4)	[37,60]
Hydropic degeneration of liver	Autopsy	1	7	2 (4)	[72,87]
Large for gestational age (LGA)	Clinical			1 (2)	[16]
Head lag	Clinical			1 (2)	[16]
Failure to gain weight	Clinical			1 (2)	[16]
Sacral dimple	Clinical			1 (2)	[16]
Congenital heart disease	Clinical			1 (2)	[16]
Dyskinesia	Clinical			1 (2)	[68]
Increased deep tendon reflexes	Clinical			1 (2)	[77]
Multiple dimples	Clinical			1 (2)	[77]
Ambiguous genitalia	Clinical			1 (2)	[72]
Coronal hypospadias	Clinical			1 (2)	[81]
Fetal macrosomia	Image			1 (2)	[16]
Abnormal umbilical artery flow	Image			1 (2)	[16]
Placental insufficiency	Image			1 (2)	[16]
Tachyarrhythmia	Image			1 (2)	[55]
Cardiomyopathy	Image			1 (2)	[55]
Hyperechogenicity of aortic valve, mitral valve, and aortic root	Image			1 (2)	[61]
Thymic calcifications	Image			1 (2)	[61]
Ascites	Image			1 (2)	[94]
Neurogenic muscle atrophy	Autopsy			1 (2)	[37]
Inflammatory infiltrate in lung	Autopsy			1 (2)	[72]
Liver apoptosis	Autopsy			1 (2)	[72]
Liver steatosis	Autopsy			1 (2)	[72]
Ventricular septal defect	Autopsy			1 (2)	[87]
