# Supplementary material for: Congenital Zika syndrome: A systematic review
Source: PLoS One. 2020 Dec 15;15(12):e0242367. doi: 10.1371/journal.pone.0242367 (PMC7737899; doi:10.1371/journal.pone.0242367)
Supplement: Attachment — Submitted filename: answer 06_07.pdf [file pone.0242367.s007.pdf]

Rio de Janeiro, July 6th 2020.

Dear Dr Jo George

Thanks for your comments.

I attached to the platform the following files.

• The supplementary file of the results of the quality assessment of components for each study included in the studies synthesis;

• The supplementary file with all strategy for searching bibliographic references. It includes all search terms and limits for at least one of the bibliographic bases;

• The supplementary file with PROSPERO registration (number: CRD42020151754)

The terms searched in PUBMED were: (((((ZIKV) OR (ZIKA)) AND (((((PREGNAN *) OR (CHILD)) OR (NEWBORN)) AND (INFANT))) AND (((((CONGENITAL) OR (CONGENITAL ABNORMALITIES)) OR (BIRTH DEFECTS)) OR (MALFORM *)) OR (MICROCEPHALY)). There was no difference in the search result, in all bases, using the mesh terms.

The searches were carried out from November 26th 2017 to December 12th 2017. 11/26/2017, 12/2/2017, 12/7/2017 and the last one on 12/12/2017.

There were no language restrictions in the searches. Besides, we included all study designs. We excluded duplicate studies and applied the inclusion and exclusion criteria.

The returns by database were: Medical Literature Analysis and Retrieval System Online (MEDLINE) (336), Latin American and Caribbean Health Sciences Literature (LILACS: 51 and SCIELO: 41), Scientific Electronic Library Online, Web of

Science (399), Excerpta Medica Database (EMBASE) (724), Scopus databases (170), and Virtual Health Library (VHL) (327).

Congenital Zika syndrome is a disease that is still being characterized, so analysis from observational studies with a limited number of participants and no measure of association were included. However, the quality of the studies was assessed.

Only four studies included in our review presentedmeasures of association. Therefore, it was not possible to perform a meta-nalysis and to assess publication bias using statistical procedures. However, to minimize publication bias, we performed comprehensive search strategy (EGGER; SMITH; DOUGLAS, 2009; HIGGINS et al., 2019). We have included this information in the body of the article (please, see line 141).

Best regards,

Danielle Amaral de Freitas
